# Protocol: The impact of integrated thematic instruction model on primary and secondary school students compared to standard teaching: A protocol of systematic review

**DOI:** 10.1002/cl2.70017

**Published:** 2024-12-21

**Authors:** Klára Barancová, Jiří Kantor, Martina Fasnerová, Zuzana Svobodová, Miloslav Klugar

**Affiliations:** ^1^ Faculty of Education Palacký University in Olomouc Olomouc Czech Republic; ^2^ Faculty of Health Sciences Palacký University in Olomouc Olomouc Czech Republic; ^3^ Faculty of Medicine Masaryk University in Brno Brno Czech Republic

**Keywords:** curriculum, education, highly effective teaching, integrated thematic instruction, integration

## Abstract

This is the protocol for a Campbell systematic review. The objectives are as follows. This systematic review will examine the impact of the Integrated Thematic Instruction (ITI) on academic attainment and other possible outcomes of primary and secondary school students compared to standard teaching. We will seek to answer the following research question: What impact does the ITI/HET teaching has on academic attainment and other possible outcomes of primary and secondary school students compared to standard teaching?

## 1 BACKGROUND

### 1.1 The problem, condition, or issue

This systematic review focuses primarily on the Integrated Thematic Instruction (ITI) model (known also as Highly Effective Teaching). This part of the protocol will define terms that have direct connection or relationship with the ITI/HET model. Terms and their definitions are given here for the sake of theoretical anchoring of the ITI/HET model. To understand the basic principles of operation and application of the model in practice, it is necessary to know the theoretical basis of the approaches described below.

#### 1.1.1 Integrated teaching

Integrated teaching is an approach to arrangement of curriculum which emphasizes its complexity, globality and holistic character (Hesová, 2011; Šafránková, 2019). Thus, integrated teaching seeks to synthesize teaching, to create close links between individual educational subjects (horizontal integration). At the same time, it emphasizes the interconnection of theoretical knowledge with the practical activities of pupils, the interconnection of teaching and learning at school with the real world, practical problems, and situations (vertical integration) (Kratochvílová, 2009; Li et al., 2020). The first step toward integration is usually the search for intersubject connections (Podroužek, 2002).

The aim of integrated teaching is to combine educational subjects or educational areas into one compact unit (Podroužek, 2002). The aim of integrated teaching can be creation of an integrated subject (an integrated subject intentionally links the educational content of several disciplines based on thematic proximity), but it is also possible to include holistic topics in educational subjects (Nguyen & Thai, 2023; Podroužek, 2002; Šafránková, 2019).

The important condition of this approach to teaching is that it is based on the so‐called integrated curriculum, not the subject curriculum. The integrated curriculum is based primarily on multilateral links in the content of the curriculum, which allow the knowledge of the world as one whole (Podroužek, 2002). ITI is therefore a certain way of integrating educational content into the system: Year‐round topic – monthly sub‐topics – weekly thematic part – daily key curriculum and tasks (Šafránková, 2019).

#### 1.1.2 Thematic teaching

It is a model coordinating the content of the curriculum in which intentional creation of multilateral links between teaching content takes place. Through a chosen topic, thematic teaching takes place across educational subjects (disciplines). Practically, this means that the teacher either assigns selected topic to teaching of different subjects and then does not have to make any adjustments to regular schedule or connects teaching of several subjects to a continuous thematic block (Ghunu, 2022). The basic theme is divided into several subtopics, which pupils in any order gradually devote themselves to. The number of subtopics, degree and extent of the context are limited only by our organizational conditions (time, number of integrated subjects, possible cooperation of teachers). Importantly, students have the opportunity to explore the topic in broader context, comprehensively and from different points of view. The goal is to learn, not to apply the acquired knowledge (Kašová, 2013). Thematic teaching, which by its very nature uses intersubject relationships, is also a form of integrated teaching. It follows from the above, that the ITI method is one of the types of thematic teaching.

#### 1.1.3 Cross‐curricular approach

A cross‐curricular approach is typical for both educational methods. Cross‐curricular approach is characterized by synthesis of knowledge, skills and understandings from various subject areas (Savage & Fautley, 2010). This approach allows students to integrate content and skills from multiple content areas into one cohesive learning experience. Through the process of teaching, the students can experience their school subjects as connected and interrelated, rather than isolated and fragmented (Rattanavich, 2013).

### 1.2 The intervention

ITI is an alternative open educational model. It is based on the knowledge of human brain functioning in the process of learning (Kolář, 2012). The model was created and described by Susan Kovalik in her 1993 book based on her work with gifted children and on research on the human brain activities (Kašová, 2013; Putra et al., 2019).

The ITI model is based on three interlocking and interdependent principles. The first principle assumes that the educator in his pedagogical activities proceeds from the latest scientific knowledge of human brain functioning. The second principle is the constant updating of the teacher's knowledge and skills. The process of learning according to this model therefore functions as both science and art at the same time (Kolář, 2012). The third principle is the creation of curriculum at the class level (the teacher develops it not only based on students' knowledge but also in accordance with their current needs) while individual needs of pupils must be fulfilled in accordance with the curriculum. Curriculum creation is the creative act of the teacher (Chumdari et al., 2018; Kolář, 2012; Olsen & Kovalik, 1995).

The model is based mainly on 1 year‐round unifying theme (key theme) and its development into branching subtopics and skills (Olsen & Kovalik, 1995). Subtopics are further divided into other parts lasting approximately one teaching week and are realized in different educational subjects (Desrinelti & Miaz, 2022; Hesová, 2011).

Such a complex approach to ITI can only be met sporadically. More often, it is a one‐day or weekly ITI (Hesová, 2011). The connection with reality is important for the processing of topics, appropriate means must be used, all elements of learning must be appropriate for the age of pupils, the topic must be beneficial, the individual sub‐topics should be constantly interconnected with the central theme and the topics should be primarily attractive for pupils (Čapek, 2015; Olsen & Kovalik, 1995). The term brain‐compatible environment (Kolář, 2012) is significant for the ITI/HET model. Kovalik sets out eight principles of a brain‐compatible learning environment. If only one of them is not observed or completely missing, then the teaching cannot be effective according to the author of the model. The principles are as follows: Absence of threats, meaningful content, choice option, reasonable time, enriched environment, cooperation, immediate feedback, perfect mastery (Olsen & Kovalik, 1995).

The teaching method project‐based learning and teaching is often associated with the ITI model in various connotations. Project‐based learning and teaching, as well as a teaching method or educational approach, combines organizational forms and work methods and can take place using multiple methods, techniques, and forms of work. It is such a way of teaching and learning, where the starting point is a meaningful and interesting task, a problem that students want and need to solve. The solution is left largely to the students. Pupils reach the result by applying their own experience and skills and several new developing activities, theoretical and especially practical, independent, and cooperative, independent of the traditional division of teaching subjects. Pupils are responsible for the progress of the work and the result itself. Pupils also participate in the assessment (Boss, 2015; Šikulová, 2007).

The ITI model and the project‐based learning and teaching method share certain characteristics. In both cases, it is an innovative and activating teaching method. Both methods are highly motivating for students and support a safe and cooperative collective climate. Teaching methods are demanding for teacher preparation. It is also crucial that these methods involve the integration of educational content and the use of interdisciplinary relationships (Arwin et al., 2021; Boss, 2015).

In addition to common characteristics, however, one can find areas where there are differences in both teaching methods. In the case of the project‐based learning and teaching method, the concentration idea is in the form of a task or problem (with ITI, a topic). The output of project‐based teaching and learning is usually a key product, a result that is known from the very beginning, whereas with ITI it is the goal of mastering the topic and the output is then smaller partial creations created during the fulfilment of application tasks. The role of the teacher in the learning process is also different for both teaching methods. In project‐based teaching, the teacher is in the position of an advisor and his role consists more in consulting or supervising the student's activities, in ITI, the teacher actively manages the activities of the students (however, he can also act as an advisor). Project teaching is challenging for teachers here in terms of flexibility in the learning process, at ITI the difficulty lies in the process of managing the teaching process. It can also be inferred from the role of teachers that a higher level of activity, independence and creativity is required of pupils during project teaching. The approach to teaching is therefore rather inductive in project teaching, in contrast to ITI, where the approach is deductive. Teacher preparation for ITI is more detailed and somewhat more demanding than in the case of project‐based teaching and learning (Lamer & Boss, 2018; Lukášová, 2010).

Even though both teaching methods share certain similar characteristics, it is clear from the above that they are significantly different teaching methods, and therefore project‐based teaching and learning will not be the subject of this systematic review.

### 1.3 How the intervention might work

The key for ITI is interconnection of educational content and creation of multilateral links between the teaching content (Kolář, 2012). In practice, application tasks are important. These tasks are designed to enable students to actually use the knowledge and skills they have learned. They should be focused practically and related to the real world. It is the students' awareness that their knowledge and skills can be useful in real life that positively affects their long‐term behaviour. Bloom's taxonomy (Čapek, 2015) is mainly used in the creation of application tasks. Teachers should also observe the following principles: The teacher's interpretation should not be longer than 11–16 min per lesson, the written examination of knowledge should be used only minimally and only where other methods of ascertaining of acquired knowledge cannot be used, the problems of real life should be deliberately solved, emphasis is placed on mutual learning of pupils and the reduction of less effective teacher interpretation (Čapek, 2015).

### 1.4 Why it is important to do this review

We understand the term pedagogical innovation as development and practical introduction of new elements into teaching to improve the teaching system. Some pedagogical innovations represent minor adjustments, others are perceived as radical changes. Many of these innovations have elements in alternative systems (Mazáčová, 2008).

Various innovation types are included in education systems around the world – whether at the level of individual learning tasks or at the level of a complete concept of teaching methods or approaches. According to different types of innovative methods and approaches to teaching, thousands if not hundreds of thousands of pupils are educated. It would therefore be appropriate to clearly determine which innovations are functional and useful for both pupils and teachers and which are not.

As mentioned above, the ITI/HET is an alternative, innovative model that has been incorporated into education systems and programmes of many countries around the world. The model was created in the 1990s in the USA specifically in California and within a few years has spread to Canada, Europe (Finland, Austria, Germany, Slovakia, etc.) (Piovarčiová, 2007), Asia (Indonesia) (Chumdari et al., 2018), Africa (Nigeria) and to other countries. There are dozens of associations around the world providing educational courses on this topic. According to the model, pupils are taught not only at primary and secondary schools, but the model can also be used for pre‐school and university education (Bírešová, 2021; Olsen & Kovalik, 1995). At the same time, the ITI/HET model is intended not only for teachers but can also be used by parents or employers (Olsen & Kovalik, 1995). Given this scope, it is surprising that there has not yet been a systematic review to verify the effectiveness of this model. The existing systematic reviews or protocols of ongoing systematic reviews were searched in Epistemonikos, Campbell Systematic Reviews, Cochrane, JBI Evidence Synthesis, and PROSPERO. The creation of this systematic review protocol is motivated by following reasons: (i) robust evidence of the effectiveness of ITI/HET is needed, (ii) factors that influence this effectiveness should be examined (due to wide international extension of ITI/HET, it is important to know, e.g., the impact of demographic factors) to provide recommendations on the use of ITI/HET for educational policy makers.

## 2 OBJECTIVES

This systematic review will examine the impact of the ITI on academic attainment and other possible outcomes of primary and secondary school students compared to standard teaching. We will seek to answer the following research question: What impact does the ITI/HET teaching has on academic attainment and other possible outcomes of primary and secondary school students compared to standard teaching?

## 3 METHODS

Studies included in the review meet the following selection criteria for study design, population, outcome and intervention (Alexander et al., 2024; Klugar, 2015; Methley et al., 2014).

### 3.1 Criteria for considering studies for this review

#### 3.1.1 Types of studies

This review will consider all quantitative primary studies and mix designed research studies that inform about the effectivity of ITI/HET. RCT studies, quasi‐experiments with control group and pre‐test and post‐test studies will be evaluated. There will be no time or language restrictions.

#### 3.1.2 Types of participants

The eligible population includes primary and secondary school students. The expected age range for students is between 6 and 20 years. There will be no restriction on school type (both regular schools and special schools will be included), gender, nationality, ethnic, racial and cultural affiliation, socio‐economic or religious status of pupils. Studies with pupils with various types of educational problems or special educational needs will also be included in the systematic review.

Studies conducted in tertiary education students (universities, higher vocational schools) and pre‐school pupils will be excluded. Deliberately, the age limit for participants was not chosen. Students may be of any age if they meet the condition of current studies at primary and secondary schools. Studies focusing on primary and secondary school teachers will not be included.

#### 3.1.3 Types of interventions

The monitored intervention for the needs of this systematic review will be the ITI model (known also as Highly Effective Teaching, ITI/HET). Studies on thematic teaching will be also considered for inclusion.

The intervention must be delivered directly to children and involve their active participation. The intervention must be used for at least 2 weeks, another requirement is the use of intervention for at least one coherent topic, followed by application into at least three educational subjects, regardless of the subject type.

If the intervention contains additional co‐intervention the study will first be included in a subgroup comprising studies that combine ITI/HET and other interventions. In the second phase, they will be grouped together in this sub‐group of studies which coincide in the type of co‐intervention. Studies focusing on the creation and application of ITI/HET study materials will be included if these studies provably measure the impact of ITI/HET on monitored outcomes. Data regarding other interventions (e.g., in multiple‐arms studies) won't be considered in this systematic review.

#### 3.1.4 Types of outcome measures

##### Primary outcomes

The primary outcome will be the academic attainment. In this systematic review, the measurement of the effects of ITI/HET on academic attainment will not be limited to one specific subject. All studies measuring academic attainment will be included in the systematic review regardless of the educational subject. Academic attainment will be measured primarily by standardized tests of academic attainment (Test of Academic Achievements, Indiana Statewide Testing for Educational Progress‐Plus), then by grades (GPA), and then by self‐constructed tests of the authors of primary studies (preference will be given to studies that report psychometric qualities of the scale constructs).

##### Secondary outcomes

Data related to any other secondary outcomes will be extracted and meta‐analysis will be performed wherever possible.

Expected secondary outcomes may include:

- attitudes values skills,
- abilities,
- motivation,
- creativity,
- interest in learning.

Based on the previous knowledge of the authors, there are not typically reported adverse events related to ITI/HET. But data concerning any harms/adverse events caused by ITI/HET will be extracted and meta‐analyze, if possible.

### 3.2 Search methods for identification of studies

#### 3.2.1 Electronic searches

The systematic review will search various electronic databases to identify studies for inclusion in the review. The search will not be limited by date of publication and language of the study.

We will search the following databases:

- Academic Search Ultimate (EBSCOhost),
- APA PsycInfo (EBSCOhost),
- ERIC (EBSCOhost),
- Web of Science Core Collection – Social Science Citation Index and Arts & Humanities Citation Index (Clarivate Analytics).

Separately we will search in:

- Campbell Systematic Reviews (Wiley Online Library).
  The terms used for the search are as follows:
  1. highly effective teaching.
  2. highly effective learning.
  3. integrated thematic instruction.
  4. integrated thematic teaching.
  5. integrated thematic learning.
  6. thematic teaching.
  7. theme based teaching.
  8. thematic approach.

The full strategy is available in Appendix S1.

Relevant and targeted subject headings will be included in search strategies, specifically at each database (thesaurus or subject index).

#### 3.2.2 Searching other resources

In addition to searching electronic databases, we will also identify studies through grey literature and through complementary search techniques explained below. We will search the following resources for grey literature:

- Google Scholar (Google),
- ProQuest Dissertation and Theses (ProQuest),
- OATD (AOTD.org),
- Open Dissertations (EBSCOhost).

In addition, we will use Google Scholar to search for references and to conduct forward citation. The titles of these included studies will be searched in Google Scholar to see how many times they have been cited and to see if any of the citing references might be relevant for the current review. Google Scholar will be searched in a private browser window to avoid any personalization of results. In Google scholar, we will search Google Scholar (manual search) and first 100 results will be considered, and the full strategy is available in Appendix S1.

A search within conference papers will not be performed. It would not be beneficial for this type of systematic review and its topic. Furthermore, we have not identified any organization or government agency that could provide information and useful documents suitable for inclusion in this systematic review.

Dissertations will be searched separately in Open Dissertations (EBSCOhost) and in ProQuest Dissertations & Theses Global (ProQuest).

Experts in the field will be identified once the included studies have been selected to see if any names appear more often. These authors will then be contacted to see if they know of additional studies that might meet this review's inclusion criteria.

### 3.3 Data collection and analysis

#### 3.3.1 Description of methods used in primary research

When collecting and analyzing data we will follow the following steps:

- Systematic and comprehensive search,
- Screen for eligibility (title, abstract, full text – first a search by abstract and title, followed by full text search),
- Data extraction,
- High‐level appraisal of systematic reviews,
- Analysis (applied inclusion and exclusion criteria) (White et al., 2020).

The study search will take place in all the above‐mentioned databases and information sources in the specified time periods according to the schedule. Records corresponding to the specified dates will be downloaded to the Rayyan citation manager (https://www.rayyan.ai/). Duplicate studies will be deleted in the next phase.

The next step will be to assess the relevance of records to the eligibility criteria. This assessment will take place in two steps. In the first step, the records will be assessed at the level of title/abstract. In the second step, the records will be assessed at the level of full texts. Screening studies in both phases will be carried out by two independent reviewers, to maintain the quality of the assessment process and reduce possible errors (Polanin et al., 2019; Stoll et al., 2019). The screeners will be blind to each other's work until comparing final judgements. Any disagreements at any stage will be resolved through discussion or with a third evaluator. The progress and results of the screening will be recorded in the PRISMA flow diagram (Page et al., 2021).

#### 3.3.2 Selection of studies

The methodological quality of RCT studies will be assessed by two independent evaluators using RoB II, for other designs the JBI risk of bias assessment tools will be used (Sterne et al., 2019). The discrepancy in the evaluation process between the two reviewers will be resolved by discussion. If there is no consensus, the third reviewer will decide on the inclusion. Studies will not be excluded based on the outcome of the critical assessment (Alexander et al., 2024; Tufanaru et al., 2020).

#### 3.3.3 Data extraction and management

Data extraction will be performed for selected studies. JBI SUMARI will be used for data extraction. Data will be extracted by two independents extractors (K. Barancová and M. Fasnerová) based on pilot data extraction stage. With data extraction, we want to prevent accidental errors during data extraction, therefore the extractors undergo a joint standardized training in which data extraction will take place for the first five studies. Any mismatch in the evaluation process between the two reviewers will be resolved by discussion (Alexander et al., 2024; Munn et al., 2019). If available, the following data listed in Table 1 will be extracted.

**Table 1:** Data extraction table.

Type	Data
Study data	Author, year of publication, country
Methodology of study	Design of study, population (age, gender), sample size (number of experimental and control group)
Intervention	Type of intervention (ITI/HET separately, co‐intervention), duration, number of topics applied, the subject in which the intervention is applied, outcome(s) measured (instrument), teachers' training, comparator
Intervention results	Pre‐intervention, post‐intervention, intervention outcome score, overall change in outcome score, and follow‐up, such data are available
Results of studies	The size of the effect for experimental and control groups

#### 3.3.4 Assessment of risk of bias in included studies

Each study included in the review will be assessed in terms of the risk of distortion by two independent reviewers. Any disagreements that arise between reviewers will be resolved by discussion or by third reviewer. The evaluation tool RoB II will be used to identify methodological quality of RCT studies. For other types of studies, the current JBI check lists for critical appraisal will be used. The choice of individual check sheets will correspond to the study type (Sterne et al., 2019; Tufanaru et al., 2020).

#### 3.3.5 Measures of treatment effect

Meta‐analysis will be performed using RevMan (RevMan Web, 2020). When meta‐analyzing the data, we will consider factors such as data variance or file size in primary studies (weighted average). We will determine both the intervention effect for each study and the weighted intervention effect for all included studies at the same time (weighted mean differences, WMD). If data from individual studies were obtained through various tests and scales that are not compatible with each other, the results will be expressed contextually to the standard deviation (standardized mean differences, SMD) (Borenstein et al., 2009). If standard deviations are missing, we will try to calculate or add them according to the instructions in the Cochrane Manual (Higgins et al., 2023; McKenzie et al., 2019).

We will assess homogeneity among studies employ using statistical model that accommodates heterogeneity among effect sizes, such as a random‐effects model. In the absence of heterogeneity, the parameter estimates of this model equal those of a fixed‐effect model. We will use both models and explore the differences.

The results of measurements in some studies, which may potentially be included in this systematic review, provide predominantly dichotomic data. For dichotomous outcomes, odds ratios can also be calculated, offering the advantage that these odds ratios can be converted into standardized mean differences (Higgins et al., 2023; McKenzie et al., 2019).

#### 3.3.6 Unit of analysis issues

In RCT studies, each study will be assessed to determine how and when randomizations occurred. This will be taken into account when considering whether participants were randomized in a cluster or group or whether they were randomized individually. If a study compared the effects of sensory interventions across two treatment groups on the outcome relative to the control, the two treatment groups will be included using robust variance estimation techniques (Hedges et al., 2010).

#### 3.3.7 Dealing with missing data

If the studies do not contain necessary complete data, their authors will be contacted to supplement the information. For the results to be eligible for inclusion in the meta‐analysis, the studies must allow numerical magnitude of the effect to be calculated. If studies lack aggregate data such as missing standard deviations or mean values, we derive them where possible, for example from *F*‐ratios, *t*‐values, *χ* ^2^ values and correlation coefficients using methods proposed by Lipsey and Wilson (2001). If these statistics are also missing, we will ask the authors of the study for information. If the missing summary data necessary to calculate the magnitude of the effect cannot be inferred or obtained, the results of the study will be presented in as much detail as possible, that is, the study will be included in the review but excluded from meta‐analysis (Sterne et al., 2016; Sterne et al., 2019).

#### 3.3.8 Assessment of heterogeneity

The heterogeneity will be evaluated using *I* ^2^ and *τ* ^2^ statistics (Deeks et al., 2022; Tanner‐Smith et al., 2016).

#### 3.3.9 Assessment of reporting biases

If 10 or more studies are included in the meta‐analysis, a funnel plot will be generated using RevMan (RevMan Web, 2020) to assess the publication bias (Sterne et al., 2011). Initially, the publication bias will be evaluated in the form of visual examination of the funnel plot and where the suitability of the use of other tests is evaluated, statistical tests on the asymmetry of the funnel plot (Egger test) will be performed. If, after evaluating the Egger test, an asymmetry of funnel surfaces is re‐detected, we will try to explain the possible reasons for the distortion (poor methodological quality leading to false overvalued effects in smaller studies, unreported distortion, and actual heterogeneity) (Egger et al., 1997).

#### 3.3.10 Data synthesis

Systematic review will take place on pre‐defined roles. We plan to do a meta‐analysis if possible. Statistical analyses will be performed using a fixed effect model where there is a low number of studies, and we will use random effects models wherever there are more than 5 studies. Data from single group observational studies will be synthetized using proportional meta‐analysis in JBI SUMARI (Munn et al., 2019). Heterogeneity will be evaluated statistically using the standard *χ* ^2^ and *I* ^2^ tests. For *χ* ^2^, *p* 0.10 (Tufanaru et al., 2015) will be used as a threshold for statistical significance.

#### 3.3.11 Subgroup analysis and investigation of heterogeneity

Studies will be divided into subgroups according to two basic criteria. The first criterion will be the type of intervention. The studies will be divided into subgroups according to the type of intervention as follows: ITI/HET without co‐intervention and ITI/HET with co‐intervention. In addition, subgroups will be divided according to the degree of education of pupils participating in the research (primary and secondary school pupils).

#### 3.3.12 Sensitivity analysis

The meta‐analysis will be carried out separately for studies with the design of RCT/quasi‐experimental studies. The robustness and generalizability of the results will be examined by various sensitivity analysis. Studies that distort results due to low methodological quality or other reasons will be excluded.

#### 3.3.13 Treatment of qualitative research

We do not plan to include qualitative research.

#### 3.3.14 Summary of findings and assessment of the certainty of the evidence

The Grading of Recommendations, Assessment, Development and Evaluation (GRADE) (GRADE Working Group, 2012) will be used to assess the degree of certainty of the evidence. A table will also be provided to summarize the results of the evidence security found. This table will be created using GRADEpro GDT (GRADEpro GDT, 2015). The assessment will be carried out by two independent evaluators. Any disagreements at any stage will be resolved through discussion or by third reviewer. A degree of certainty rating ranging from high, medium, low to very low will be assigned to the body of evidence (Schünemann et al., 2013).

## CONTRIBUTIONS OF AUTHORS

- Content: Klára Barancova
- Systematic review methods: Jiří Kantor, Fasnerová Martina
- Statistical analysis: Jiří Kantor, Miloslav Klugar
- Information retrieval: Zuzana Svobodová

## DECLARATIONS OF INTEREST

No conflict of interest detected.

## PRELIMINARY TIMEFRAME

We plan to submit a draft review: 1 September 2024.

## PLANS FOR UPDATING THIS REVIEW

Klara Barancova will be primarily responsible for updating this review. She will update the content every 2 months.

## SOURCES OF SUPPORT

### 1 Internal sources

IGA_PdF_2023_001, Czech Republic IGA – Competition to support projects of specific university research carried out by students of a doctoral or master's study programme at the Palacký University Olomouc.

### 2 External sources

No sources of support provided.

## Supporting information

Supporting information.
