# Supplementary material for: Protocol: The impact of integrated thematic instruction model on primary and secondary school students compared to standard teaching: A protocol of systematic review
Source: Campbell Syst Rev. 2024 Dec 21;20(4):e70017. doi: 10.1002/cl2.70017 (PMC11663231; doi:10.1002/cl2.70017)
Supplement: Supplementary file 1 — Supporting information. [file CL2-20-e70017-s001.docx]

# Appendices

##### Appendices should be submitted as [supplementary material](https://authorservices.wiley.com/author-resources/Journal-Authors/Prepare/manuscript-preparation-guidelines.html/supporting-information.html).

## 1 Example - Search strategies:

Academic Search Ultimate - Search was conducted on the 28th May 2024 10:42-11:04 (CET)

| **#** | **Searches** | **Results** |
| --- | --- | --- |
| 1 | DE[1] „THEMATIC approach in education“ OR DE „EFFECTIVE teaching“ OR “highly effective teaching” [Title/Abstract] OR “highly effective learning*” [Title/Abstract] OR “integrated thematic instruction*” [Title/Abstract] OR “integrated thematic teaching” [Title/Abstract] OR “integrated thematic learning” [Title/Abstract] OR “thematic teaching” [Title/Abstract] OR “theme based teaching” [Title/Abstract] OR “thematic approach*” [Title/Abstract] OR „thematic learning“ [Title/Abstract] | 10,589 |
| 2 | DE „STUDENTS “OR DE „CHILDREN“OR “pupil$” [Title/Abstract] OR “young*” [Title/Abstract] OR “child*” [Title/Abstract] OR “student*” [Title/Abstract] OR “teenage*” [Title/Abstract] OR “adolescent*” [Title/Abstract] OR “juvenile” [Title/Abstract] OR “teen$“ [Title/Abstract] | 3,164,043 |
| 3 | “elementary education “[Title/Abstract] OR „elementary school*“[Title/Abstract] OR “secondary school*“ [Title/Abstract] OR “primary education“ [Title/Abstract] OR secondary education“ [Title/Abstract] OR „primary school*“ [Title/Abstract] OR DE "ELEMENTARY Education" OR DE "ELEMENTARY Schools" OR DE "SECONDARY Education" OR DE „SECONDARY Schools“ | 150,527 |
| 4 | #1 AND #2 AND #3 | 651 |

APA PSycINFO - Search was conducted on the 28th May 2024 11:04-11:10 (CET)

| **#** | **Searches** | **Results** |
| --- | --- | --- |
| 1 | “highly effective teaching” [Title/Abstract] OR “highly effective learning*” [Title/Abstract] OR “integrated thematic instruction*” [Title/Abstract] OR “integrated thematic teaching” [Title/Abstract] OR “integrated thematic learning” [Title/Abstract] OR “thematic teaching” [Title/Abstract] OR “theme based teaching” [Title/Abstract] OR “thematic approach*” [Title/Abstract] OR „thematic learning“ [Title/Abstract] | 882 |
| 2 | DE „STUDENTS“ OR “pupil$” [Title/Abstract] OR “young*” [Title/Abstract] OR “child*” [Title/Abstract] OR “student*” [Title/Abstract] OR “teenage*” [Title/Abstract] OR “adolescen*” [Title/Abstract] OR “juvenile” [Title/Abstract] OR “teen$“ [Title/Abstract] | 1,638,696 |
| 3 | “elementary education“ [Title/Abstract] OR „elementary school*“ [Title/Abstract] OR “secondary school*“ [Title/Abstract] OR “primary education“ [Title/Abstract] OR secondary education“ [Title/Abstract] OR „primary school*“ [Title/Abstract] OR DE "ELEMENTARY Education" OR DE "ELEMENTARY Schools" OR DE "SECONDARY Education" | 88,201 |
| 4 | #1 AND #2 AND #3 | 24 |

ERIC - Search was conducted on the 28th May 2024 11:10-11:24 (CET)

| **#** | **Searches** | **Results** |
| --- | --- | --- |
| 1 | DE „THEMATIC approach“ OR highly effective teaching” [Title/Abstract] OR “highly effective learning*” [Title/Abstract] OR “integrated thematic instruction*” [Title/Abstract] OR “thematic teaching” [Title/Abstract] OR “ thematic learning” [Title/Abstract] OR “thematic teaching” [Title/Abstract] OR “theme based teaching” [Title/Abstract] OR “thematic approach*” [Title/Abstract] OR „thematic learning“ [Title/Abstract] | 2,896 |
| 2 | DE „STUDENTS“ OR DE „CHILDREN“ OR “pupil$” [Title/Abstract] OR “young*” [Title/Abstract] OR “child*” [Title/Abstract] OR “student*” [Title/Abstract] OR “teenage*” [Title/Abstract] OR “adolescen*” [Title/Abstract] OR “juvenile” [Title/Abstract] OR “teen$“ [Title/Abstract] OR DE "Adolescents" | 1,144,353 |
| 3 | Elementary secondary education [Subject Heading] OR  elementary education [Subject Heading] OR primary education [Subject Heading] OR secondary education [Subject Heading] OR schools [Subject Heading] OR elementary schools [Subject Heading] OR junior high schools [Subject Heading] OR high schools [Subject Heading] OR middle schools [Subject Heading] OR private schools [Subject Heading] OR public schools [Subject Heading] OR secondary schools [Subject Heading] OR students [Subject Heading] OR elementary school students [Subject Heading] OR secondary school students [Subject Heading] OR junior high school students [Subject Heading] OR middle school students [Subject Heading] OR high school students [Subject Heading] OR (school* OR class OR classses OR classroom*) [Title/Abstract] OR (((school* OR elementary OR primary OR secondary OR grade) [Title/Abstract] adj3 (student* OR children)) OR pupil*) [Title/Abstract] | 1,017,089 |
| 4 | #1 AND #2 AND #3 | 1,618 |
